# Quantitative, Temperature-Calibrated and Real-Time Glucose Biosensor Based on Symmetrical-Meandering-Type Resistor and Intertwined Capacitor Structure

**DOI:** 10.3390/bios11120484

**Published:** 2021-11-28

**Authors:** Yangchuan Ma, Tian Qiang, Minjia Gao, Junge Liang, Yanfeng Jiang

**Affiliations:** Department of Electronic Engineering, School of Internet of Things Engineering, Jiangnan University, Wuxi 214122, China; 6201924123@stu.jiangnan.edu.cn (Y.M.); 6201924078@stu.jiangnan.edu.cn (M.G.); jgliang@jiangnan.edu.cn (J.L.); yanfeng_jiang@yahoo.com (Y.J.)

**Keywords:** biosensor, microfluidic channel, symmetrical meandering type resistor, intertwined capacitor, temperature calibration

## Abstract

Here, we propose a glucose biosensor with the advantages of quantification, excellent linearity, temperature-calibration function, and real-time detection based on a resistor and capacitor, in which the resistor works as a temperature sensor and the capacitor works as a biosensor. The resistor has a symmetrical meandering type structure that increases the contact area, leading to variations in resistance and effective temperature monitoring of a glucose solution. The capacitor is designed with an intertwined structure that fully contacts the glucose solution, so that capacitance is sensitively varied, and high sensitivity monitoring can be realized. Moreover, a polydimethylsiloxane microfluidic channel is applied to achieve a fixed shape, a fixed point, and quantitative measurements, which can eliminate influences caused by fluidity, shape, and thickness of the glucose sample. The glucose solution in a temperature range of 25–100 °C is measured with variations of 0.2716 Ω/°C and a linearity response of 0.9993, ensuring that the capacitor sensor can have reference temperature information before detecting the glucose concentration, achieving the purpose of temperature calibration. The proposed capacitor-based biosensor demonstrates sensitivities of 0.413 nF/mg·dL^−1^, 0.048 nF/mg·dL^−1^, and 0.011 pF/mg·dL^−1^; linearity responses of 0.96039, 0.91547, and 0.97835; and response times less than 1 second, respectively, at DC, 1 kHz, and 1 MHz for a glucose solution with a concentration range of 25–1000 mg/dL.

## 1. Introduction

A biosensor is an instrument that is sensitive to biological substances and converts their concentration into electrical signals for detection. It uses immobilized biologically sensitive materials as identification elements, including enzymes, antibodies, antigens, microorganisms, cells, tissues, nucleic acids, etc. A biosensor device or system combines appropriate physical/chemical transducers with signal amplification [1,2,3]. In regard to a glucose biosensor, the detection sample is focused on the concentration of a glucose solution. Real-time and early detection of glucose concentration has become significant in clinical diagnoses and for assessing treatment progress. Diabetes is a global health issue affecting millions of people [4]. Diabetes is closely related to insulin concentration and glucose levels must be monitored often to avoid complications caused by blood sugar fluctuations [5]. Diabetic macroangiopathy and atherosclerosis due to diabetes can cause cerebrovascular, ischemic heart, peripheral arterial, and other vascular diseases, which are important causes of death in diabetic patients [6].

Currently, more and more researchers have become interested in glucose biosensors due to the need for early detection of glucose levels. On the basis of the physical/chemical transducers of a biosensor, different types of biosensors have been achieved, such as electrochemical, optical, field effect transistor (FET), piezoelectric, microwave, and capacitor biosensors. Among them, an electrochemical biosensor realizes the selective detection of biomarkers by adding specific enzymes to electrodes [7,8]. The electrodes can be used to obtain the voltage–current curves and resistance of a tested solution at different concentrations [9,10]. An electrochemical biosensor is simple to use and cost effective [11]; however, there are still some shortcomings, such as the introduction of external media that slows the sensor’s response and decreases its reliability, and the need to replace it in a cycle of about half a year depending on the use environment. In addition, another factor restricting the application of electrochemical sensors is that the electrolyte needs to be replenished regularly and testing tips are not reusable, which may lead to an extra cost. In regard to optical biosensors, non-contact and non-destructive measurement methods can be realized based on optical principles [12,13], and they have excellent color recognition performance for test solutions added with fluorescent markers [14,15]. However, the measurement system constituted by optical devices is relatively complicated, and normal detection usually requires a long calibration and stabilization time, and it is susceptible to the influence of ambient light that causes detection errors. In regard to FET biosensors, the use of specific nanomaterials makes an FET biosensor highly sensitive, selective, and it has commercial potential [16,17]. It can be used to detect various biological or chemical molecules and to detect protein molecules as well as other ions in the physiological environment [18,19]. Nevertheless, FET biosensors are commonly fabricated using an oxide as the sensing layer. This causes the sensor to have monotonic drift and, at the same time, the response will also cause fluctuations. The use of active devices such as logic gates and transistors will increase energy consumption and will have high performance requirements for the FET chips. In regard to piezoelectric biosensors, although the sensitivity of piezoelectric biosensors is relatively high. It can detect small physiological changes similar to sweat, and the piezoelectric signal containing the change information in the concentration of a small molecule can also be obtained [20,21]; however, the selectivity and linearity of this kind of sensor still need to be improved. It is still difficult to distinguish small changes in a substance, and whether they cause discomfort in the human body remains to be discussed. In regard to microwave biosensors, they can detect and characterize the dielectric properties in materials with high Q-factors, narrow resonance, low insertion loss, and high sensitivity [22,23]. Microwave biosensors can translate a variation in dielectric properties of adjacent materials into quantifiable electrical signals such as resonant frequency and resonant amplitude in a remote non-contact manner [24]. However, a lack of accurate model characterization has restricted the simulation of microwave biosensors; the amount, position, and shape of a tested biomarker sample are important issues for accurate and quantitative detection of the biomarker solution, let alone the ambient temperature influence on detection. Recently, capacitor sensors have been especially applied for detecting humidity with high sensitivity, short response/recovery time, and good thermal stability [25,26,27]. As compared with other types of humidity sensors, it is very competitive. In addition, a capacitor-based biosensor could be a promising candidate in the biosensor research field due to its quick response time, real-time detection, excellent design flexibility, compact chip dimension, cost effectiveness, easy measurement process, convenient integration with matching circuits, etc.

In this study, we propose a resistor-based temperature sensor and a capacitor-based biosensor which can measure the temperature and concentration of a biomarker solution in real time. The concentration range of the glucose solution is 25–1000 mg/dL, which is injected into the microfluidic cavity and placed on the top of the sensors. Then, the signal is provided through an LCR meter, while capacitance and resistance are recorded. The proposed biosensor applies a polydimethylsiloxane (PDMS) quantitative cavity structure with fixed volume and fixed test points, which is convenient for quantitative detection of glucose solution samples, and can eliminate the interference and influence caused by the fluidity, shape, and thickness of glucose solution samples during the test, and therefore achieves accurate measurements of the biomarker solution. Moreover, the amount of solution required for the measurements is quite small and only 1.806 μL of glucose solution is required to complete a measurement. Commonly used and low-cost medical syringes can be used to transfer and inject the biomarker solution for measurement, without using expensive and strict quantitative pipettes. The sensor response of the proposed work is real time, i.e., after the glucose solution is introduced, the resistance and capacitance can be directly affected in less than 1 s, and the value can be read out immediately through a low-cost LCR meter instead of an expensive and complex vector network analyzer. The concentration range of the glucose solution tested in this study includes the normal range of diabetic patients currently being tested in clinical practice. 

## 2. Fabrication Process

**Figure 1:**
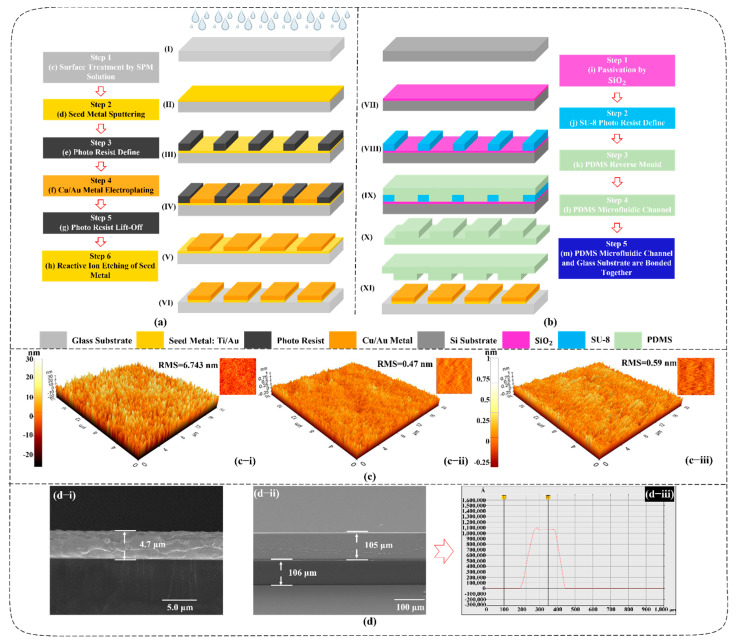
(**a**) Fabrication process of the metal structure for the resistor and capacitor; (**b**) fabrication process of the PDMS microfluidic channel; (**c**) morphological analysis; (**c–i**) surface profile of bare glass substrate; (**c–ii**) seed metal; (**c–iii**) seed metal after O_2_ etching; (**d**) morphological analysis; (**d–i**) SEM image of metal on glass substrate; (**d–ii**) SU-8 line pattern on Si substrate; (**d–iii**) height and width measurements of the SU-8 line pattern.

The proposed biosensor is fabricated on a glass substrate by micro-/nano processing technology, as shown in Figure 1. To begin, before the growth of the metal structure on the glass substrate, an atomic force microscope (AFM) image of bare glass substrate is analyzed, as shown in Figure 1(c−i). A root mean square (RMS) value of 6.74 nm is obtained, which is not good enough for the following seed metal to stick on the surface of glass, because metal that locates on a glass surface usually suffers from a peel-off issue due to the self-tension of the seed metal. Therefore, in the case of such an issue, first, the glass substrate is polished with an SPM solution (sulfuric/peroxide mi) in order to remove impurities and organic pollutants and to enhance the roughness of the surface, targeting to improve the adhesion of the following metal growth. Then, the seed metal layer of Ti/Au with a thickness of 20/80 nm is prepared by the sputtering method. An RMS value of 0.47 nm is obtained for the surface morphology of the Ti/Au seed metal, as shown in Figure 1(c−ii). In order to further ensure adhesion between the seed metal and the following photoresist as well as electroplated metal, the O_2_ etching method is applied on the surface of the seed metal and an improvement of 0.12 nm RMS value is achieved, as shown in Figure 1(c−iii). Following this, we spin a 6 μm thick photoresist on the seed metal, form the designed pattern through a photomask, and then develop the place where we are going to make the target metal through a lift-off machine. Subsequently, a 5 μm thick Cu/Au metal layer is electroplated at the notch. Finally, all photoresists are washed out with acetone solution, and the surplus seed metal layer is etched through the reactive ion etching method, the metal part of the sensor is finished without any peel-off issues between the glass surface and seed metal, between the seed metal and electroplated metal, and between the seed metal and photoresist. A scanning electron microscope (SEM) image of the cross-section of metal is demonstrated in Figure 1(d−i) with a thickness of 4.7 μm. A decrease in thickness occurs mainly due to the control tolerance of electroplating time and reactive ion etching during the isolation process of the seed metal. The details of the fabrication process of the metal structure for the resistor and capacitor are shown in Figure 1a. The fabrication of the PDMS quantitative cavity is based on a reverse molding process using a SU-8 negative photoresist on silicon substrate. Firstly, the silicon substrate is cleaned by acetone. Then, a 200 nm thick silica (SiO_2_) passivation layer is deposited over the silicon substrate using plasma-enhanced chemical vapor deposition. Next, we spin a SU-8 photoresist on the passivation layer and form the required SU-8 structure through the photomask, and then wash away the excess SU-8 photoresist through developer. The SEM image shows the SU-8’s line pattern on the Si substrate, as shown in Figure 1(d−ii). Its height and width are measured through a surface profiler; a height of approximately 105 μm can be obtained along with a width of approximately 106 μm, as shown in Figure 1(d−iii). Finally, the PDMS microfluidic channel is obtained by a reverse molding process after the SU-8 and PDMS are separated, as shown in Figure 1b. The biosensor and PDMS quantitative cavity are bonded together after the plasma bonding process and the thermal treatment to complete the whole fabrication. When the glucose solution is put into the PDMS quantitative cavity, the solution will directly contact the resistor and capacitor to change the resistance and capacitance.

## 3. Method and Analysis

A complete biosensor system should be comprised of four parts, which are the substance, as well as the detection, transduction, and signal conditioning components [28]. We are mainly focused on the transduction components based on our research background on RF/microwave engineering and micro-/nano fabrication technology for research in the field of potential glucose biosensing applications. The operation mechanism for the proposed work is on the basis of the RF/microwave theory and realistic glucose biosensing application. We describe a new concept for transducers with microfluidic devices. Our design is mainly composed of three parts, which are a temperature sensor, glucose biosensor, and a PDMS microfluidic channel.

Ambient temperature is an important factor that influences the properties of a glucose solution, especially the dielectric property. The dielectric property, which is the complex relative permittivity (ε_s_), is the key factor for the capacitor or microwave biosensor, and a variation in the dielectric property could result in a capacitance or frequency change in the biosensor. The mathematical equation is given below:

(1) $$\varepsilon_{s}=\varepsilon_{s}^{\prime }-j\varepsilon_{s}^{\prime \prime }$$

Note that, as the temperature of the glucose solution increased, the imaginary part of the complex relative permittivity of glucose $\varepsilon_{s}^{\prime \prime }$ decreases more rapidly than that of real part $\;\varepsilon_{s}^{\prime }$ [29]; the real and imaginary parts of the dielectric constant of glucose solution at 5 GHz can vary from 78.0 to 76.2 and from 15.7 to 12.4, respectively, when the ambient temperature is changed from 32 °C to 42 °C. Even for a microwave biosensor in [30], temperature is considered to be a critical parameter to scrutinize for glucose sensing application. Temperature variations from 10 °C to 50 °C have been carried out to study the temperature effect on resonating frequency for different glucose concentrations. Therefore, temperature information is needed so that the concentration of the glucose solution is tested with calibration of the ambient temperature influence.

**Figure 2:**
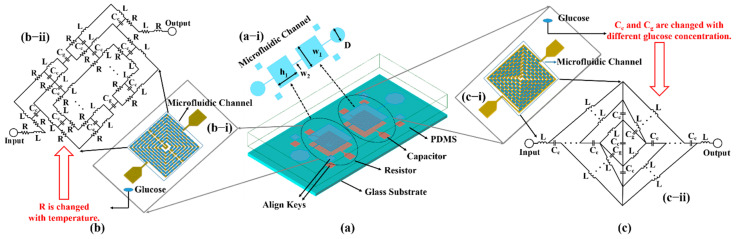
(**a**) Layout of the designed sensor: (**a–i**) shows the PDMS microfluidic channel structure with dimensions in mm, i.e., D = 2.6, w_1_ = 4.3, w_2_ = 0.1, and h_1_ = 4.2. (**b**) The temperature sensor based on resistor structure: The inset image (**b–i**) shows the microfluidic channel inserted with glucose solution; the inset image (**b–ii**) demonstrates the equivalent circuit of the temperature sensor. (**c**) The glucose biosensor based on capacitor structure: The inset image (**c–i**) shows the microfluidic channel inserted with glucose solution; the inset image (**c–ii**) demonstrates the equivalent circuit of glucose biosensor.

In this study, based on a resistor and capacitor structure, a temperature sensor and a glucose biosensor are proposed. In order to increase the contact area with the glucose solution, a symmetrical meandering type resistor and an intertwined capacitor are designed, as shown in Figure 2. Figure 2a shows the layout model established using an advanced design system; both sensors are integrated on a glass substrate with a dimension of 19.4 mm × 8.0 mm × 4.6 mm. The integrated microfluidic channel is demonstrated in Figure 2(a−i) with detailed dimensions. Figure 2(b−i) and (c−i) show the detailed schematics of the capacitor and resistor inserted with the glucose sample, separately. They all adopt the structure of a winding line, which can achieve a high number of turns in a compact area so that more inter-turn gaps can be formed, laying the foundation for a highly sensitive sensing response with the biomarker solution. The metal structure for the resistor and capacitor includes an input and an output port, respectively, two 1 mm × 1 mm align key modules. The input and output ports are used for the probe measurement of the LCR meter. An align key module is used for precise alignment when bonding with the quantitative channel structure of the PDMS, since R can change with a change in ambient temperature, resulting in total resistance variation in the equivalent circuit of the resistor-based temperature sensor, as shown in Figure 2(b−ii). Similarly, C_c_ and C_g_ can change with different glucose concentrations, resulting in total capacitance variation in the equivalent circuit of the capacitor glucose biosensor, as shown in Figure 2(c−ii).

The equivalent resistance of the resistor can be calculated using the equation below [31]:

(2) $${R=R}_{\mathrm{sh}}\frac{\left[h\cdot N+d\left(N+1\right)+2\left(b+a\right)\right]}{w}$$

where R_sh_ is the sheet resistance of the metal layer used for the resistor design; h represents the width of the winding line resistor; N represents the order of the winding line resistor; d represents the gap of the winding line resistor; a and b represent the size of the winding line at the input and output ports, respectively. The resistance is affected by the temperature of the glucose solution. When the temperature rises, due to an increase in the outermost electron energy of the atom, it can move irregularly and freely, so that the metal can conduct electricity. However, in addition to the free electrons, the atoms in the metal also vibrate. The higher the temperature, the stronger the vibration, which increases the probability of collision between the free electrons and atoms, hinders the directional movement of electrons, and leads to an increase in metal resistance, that is, the value of R_sh_ increases, and metal commonly performs a positive temperature coefficient at a certain temperature range. The relationship between the resistance and temperature is given as follows [32]:

(3) $$R\left(T\right){=A+\mathrm{Ce}}^{\mathrm{BT}}$$

where T is temperature (K); R(T) is the resistance (Ω); and A, B, and C are constants whose values are determined by conducting experiments at two temperatures and solving the equations simultaneously. As we calculated based on Equation (3), the resistance increases exponentially with increasing temperature for a metal with a positive temperature coefficient, therefore we use Cu/Au to fabricate the resistor structure and take advantage of a fraction of the exponential curve at a certain temperature range, in which the curve almost performs similar to a linear characteristic. The relationship between resistance and glucose solution temperature can be used to perform the temperature sensor response.

The capacitance of the biosensor is affected by the concentration of glucose solution, the equivalent capacitance of the capacitor can be calculated using the equation below [33]:

(4) $$C=\left[\varepsilon_{0}\varepsilon_{\mathrm{sub}}\left(\frac{{1+\varepsilon }_{s}}{2}\right)\frac{K\sqrt{{1\;-\;k}^{2}}}{K\left(k\right)}{+\varepsilon }_{0}\frac{t}{a}+{\left\{\frac{K\left(k\right)}{\varepsilon_{0}K\sqrt{{1\;-\;k}^{2}}}\right\}}^{-1}\right]L_{C}$$

where L_c_ is the coupled meandered-line length, t is the thickness of the metal, a is the distance between the winding line resistors, ε_0_ (equals to 8.854 × 10^−12^) denotes the free space permittivity, ε_s_ denotes the permittivity of the glucose solution, and ε_sub_ (equals to 4.1) denotes the permittivity of glass substrate; k (=a/b) and K(k) are the elliptic integrals of the first kind. Since the viscous effect increases as the concentration of the glucose solution increases, resulting in increased relaxation times and correspondingly decreased dielectric constants and increased loss factor [34,35], as a consequence, the capacitance of the proposed sensing capacitor in this study is expected to be maximized and minimized when the glucose level in the glucose sample is minimized and maximized at 25 mg/dL and 1000 mg/dL, respectively. Therefore, the relationship between capacitance and glucose concentration can be used to perform the biosensor response.

The equivalent circuits of the biosensor structures are depicted in Figure 2b,c, respectively. They represent the series/parallel combination of the inductor and capacitor or resistor which are equivalent to electrodes’ conductive paths and the gap between them. In the presented equivalent structures, L represents inductance, R represents resistance, C_g_ represents gap capacitance, and C_c_ represents coupling capacitance. In the equivalent circuit of the resistor, because input and output ports are connected with each other, the inductor and resistor play the main role in the circuit, and the influence of coupling capacitance can be ignored. At the same time, because temperature has a significant influence on the resistance of metal, the resistor can be used to calibrate the temperature of the glucose solution in this design. In the equivalent circuit of the capacitor, because the input and output ports are disconnected, the inductor and capacitor play the main role in the circuit, and the influence of resistance can be ignored. When the glucose solution is introduced into the PDMS microfluidic channel, the solution fills all gaps, and the dielectric constant of the glucose solution with different concentrations are different, which will affect the coupling capacitance and gap capacitance. Therefore, in this design, the capacitor can be used to measure the concentration of the glucose solution. Moreover, the PDMS microfluidic cavity is designed to ensure that the glucose solution is in full contact with the metal structure at a fixed position and a fixed shape, so that the error caused by the different positions can be avoided and the error of solution quantity caused by the direct dropping can be eliminated. The inset image, as shown in Figure 2(a−i), shows the structure and dimension of the PDMS microfluidic channel. The height of the cavity is 0.1 mm, and the depth of the whole PDMS is 3.8 mm. The quantitative cavity structure includes a capacitor cavity and a resistor cavity, which are connected by a microchannel with a height of 0.1 mm and a width of 0.1 mm. A through hole with a diameter of 2.6 mm can be obtained at each end of the channel through a hole punch, separately, which is prepared for the joint with the plastic hose in the later experiment, so as to ensure a complete fit with the plastic hose without liquid leakage. Furthermore, there are four calibration modules around the PDMS quantitative cavity structure, which correspond to the metal structure alignment modules, respectively.

## 4. Experimental Setup

**Figure 3:**
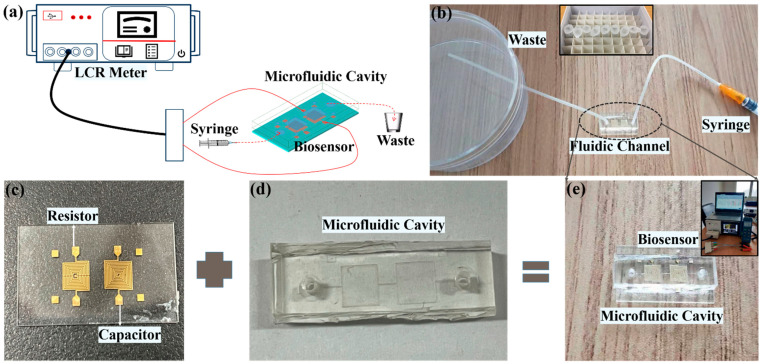
(**a**) Schematic diagram of the sensor and experimental setup; (**b**) measurement view of the solution injected into the microfluidic channel, the inset image is glucose sample; (**c**) fabricated sensor with metal structure; (**d**) fabricated PDMS microfluidic channel; (**e**) proposed sensors integrated with microfluidic channel, inset image is the LCR meter that records the experimental data.

The proposed biosensor is fabricated on a glass substrate with a dielectric constant and loss tangent of 4.1 and 0.08, respectively. The thicknesses of the glass substrate and metal layer are 0.8 mm and 5 μm, respectively. The glucose solution interacts with the biosensor through the PDMS microfluidic cavity. An LCR meter (HIOKI IM3536) is used to measure the capacitance and resistance as the sensor response. Figure 3a shows the schematic diagram of the test setup. After the LCR meter is connected to the sensor, when the glucose solution is put into the PDMS microfluidic cavity, the changes in resistance and capacitance of the sensor can be observed. Such a measurement is completed only with a solution volume of 1.806 μL, which is cost effective for the tested biomarker solution.

In order to verify the experimental results, we used a glucose sample to measure the sensor response. The overall measurement process is shown in Figure 3b, after connecting the two interfaces of the LCR meter to both ports of the capacitor or resistor. A syringe is used to inject the biomarker solution into the tube and the PDMS microfluidic channel. Then, the glucose solution is in contact with the resistor and capacitor in the microfluidic cavity. At the same time, the LCR meter can display the resistance and capacitance in real time. Finally, the used solution is stored in a beaker after passing through the biosensor. In this experiment, the glucose solution is used with concentrations of 25, 50, 100, 200, 300, 400, 500, 600, 800, and 1000 mg/dL, as shown in Figure 3b. They are prepared using a quantized mixture of D-(+)-glucose powder (Aladdin Bio-Chem Technology Co., LTD) and deionized (DI) water. The fabricated sensor is shown in Figure 3e, which mainly includes three parts: the glass substrate, the metal structure of the sensor, and the PDMS quantitative cavity structure. The glass substrate has the advantages of smooth surface, easy PDMS bonding property, and transparency, which make it a strong candidate for a microfluidic-based application, as shown in Figure 3c. The metal structure for the resistor and capacitor is designed with a single layer structure which is easy to process and low in cost. The metal structure for the resistor and capacitor at high frequency is more easily affected by the concentration of the biomarker solution, the structure is shown in Figure 3c. The PDMS materials have uniform texture, few bubbles, strong bonding with glass, easy molding with the SU-8 photoresist mold, soft texture, and they are easy to combine with flexible electronic applications. The PDMS material after the plasma bonding process can be firmly and tightly combined with the glass substrate, and the area where the measurement port is located is reserved around the PDMS material, as shown in Figure 3d. An LCR meter is used to input signals of different frequencies and measures the corresponding capacitance and resistance, as shown in Figure 3e. During the experiment, the same concentration of glucose solution is measured three times for temperature sensor measurements and biosensor measurements, separately. After each measurement, another syringe is used to inject DI water to clean the glucose solution, and air is injected through different syringes to dry the DI water. Before each measurement, it should be ensured that there is no residual solution in the biosensor module, and the channel is dry and free from blockage.

## 5. Results and Discussion

**Figure 4:**
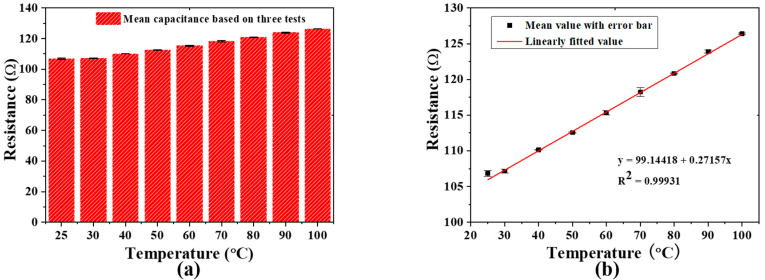
(**a**) The average value of the resistor biosensor response to the glucose solution at different temperatures; (**b**) regression analysis of resistance (n = 9) at different temperatures.

We injected glucose solutions with different concentrations into the microfluidic cavity and recorded the response of the temperature sensor with an LCR meter. A remarkably quick response time of less than 1 s was realized when the tested glucose was injected into the shape-fixed, position-fixed, and volume-fixed PDMS microfluidic structures, which is shown in the supplementary video in Supplementary Materials. Next, we measured the resistance using a temperature sensor. Figure 4a shows the resistance measurement histogram of the glucose solution in the temperature range of 25–100 °C. Figure 4b shows the linear relationship between the temperature of the glucose solution and the change in resistance realized by the resistor-based temperature sensor. This relationship can be expressed as:

y_1_ [Ω] = 99.14418 + 0.27157 × x [°C], a good correlation with the linear fit of R^2^ = 0.99931, relative standard deviation (RSD) ≦0.49558, sensitivity is 0.272 Ω/°C, where y_1_ is the resistance of the resistor-based temperature sensor, and x is the temperature of the glucose solution. The regression analysis data show that the temperature of the glucose solution has a good linear correlation with the resistance of the resistor-based temperature sensor, that is, it can measure the temperature of the glucose solution before the capacitor-based biosensor measures the concentration, and therefore assists in calibrating the influence of solution temperature.

Under the condition of ambient relative humidity ranging from 47.4% to 48.6%, the capacitances of the capacitor-based biosensor for bare chip and DI water are measured three times under different input frequencies, as shown in Table 1. It can be seen that with an increase in input frequency, the measured capacitances show a decreasing trend in the above two cases. Such a phenomenon is mainly caused by a decrease in the effective dielectric constant of the glucose solution when the input frequency increases [35]. The measured capacitances of the bare-chip capacitor at DC, 1 kHz, and 1 MHz are far different from the capacitance measured after passing glucose solution. Therefore, in the actual measurements, the influence of bare chip and DI water on the concentration of glucose solution can be ignored.

**Figure 5:**
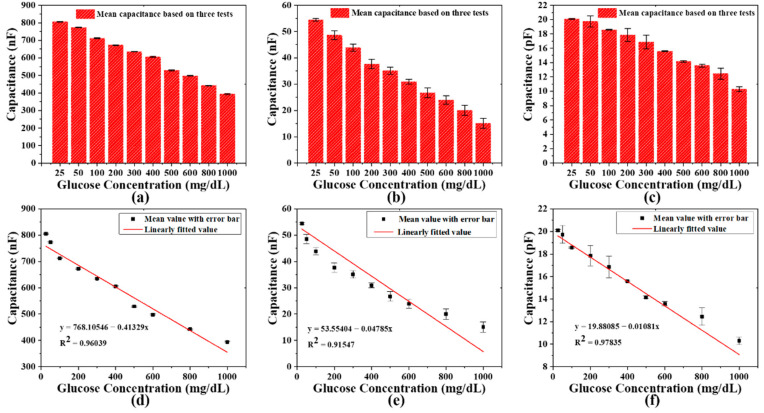
The average of three sensing responses of the capacitor-based biosensor to different concentrations of glucose solution at: (**a**) DC signal frequency; (**b**) 1 kHz signal frequency; (**c**) 1 MHz signal frequency. Regression analysis is performed for different glucose concentrations (n = 10) at: (**d**) DC signal; (**e**) 1 kHz signal; (**f**) 1 MHz signal.

The measured sensing responses of the capacitor-based biosensor to different concentrations of glucose solution are shown in Figure 5. Figure 5a–c, respectively, shows the measurement histograms of the glucose solution in the concentration range of 25–1000 mg/dL based on the capacitor-based biosensor at DC, 1 kHz, and 1 MHz signal frequencies. Figure 5d–f shows the linear relationship between the glucose solution concentrations and capacitance change realized by the capacitor-based biosensor with input signals of DC, 1 kHz, and 1 MHz. The corresponding linear regression equations are as follows:

y_2_ [nF] = 768.10546 − 0.41329 × x [mg/dL], a correlation with the linear fit of R^2^ = 0.96039, RSD ≦ 2.63909%, sensitivity is 0.413 nF/mg·dL^−1^;

y_3_ [nF] = 53.55404 − 0.04785 × x [mg/dL], a correlation with the linear fit of R^2^ = 0.91547, RSD ≦ 1.97151%, sensitivity is 0.048 nF/mg·dL^−1^;

y_4_ [pF] = 19.88085 − 0.01081 × x [mg/dL], a correlation with the linear fit of R^2^ = 0.97835, RSD ≦ 0.96428% sensitivity is 0.011 pF/mg·dL^−1^; where y_2_, y_3_ and y_4_ are the capacitances of the capacitor-based biosensor at DC, 1 kHz, and 1 MHz; x is the concentration of glucose solution in the range of 25–1000 mg/dL. The regression analysis reveals a good linear correlation between glucose concentration and capacitance. In addition, it shows that the capacitance can be used as a parameter for glucose concentration detection, and the limit of detection (LOD) values of the capacitor-based biosensor are 67.236 mg/dL, 17.724 mg/dL, and 2.944 mg/dL at the input signal frequencies of DC, 1 kHz, and 1 MHz, respectively. The RSD of the mathematical parameters can be obtained based on the statistical data of multiple measurements of the capacitance, which are less than 3%, 2%, and 1% at the signal frequencies of DC, 1 kHz, and 1 MHz, respectively, indicating that the capacitance of the capacitor-based biosensor is quite stable in the process of multiple measurements.

**Figure 6:**
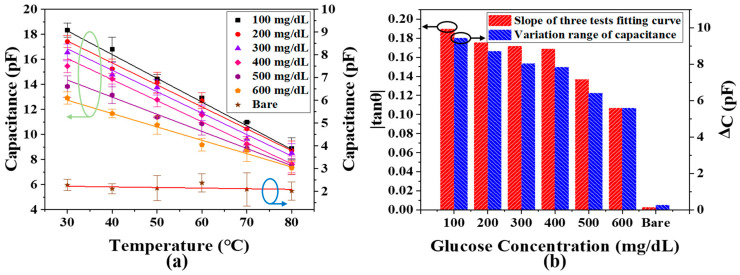
(**a**) The response of the bare chip and capacitor to different concentrations of glucose solution at the temperature range of 30–80 °C; (**b**) the change of slope for the fitting curve between different concentrations of glucose solution and capacitance, and the change range of capacitance with temperature.

Since environmental temperature is a key factor that influences the measurement accuracy of a glucose biosensor, we performed a temperature calibration test based on the 1 MHz capacitive biosensor and measured the sensing response at a temperature range between 30 °C and 80 °C. As shown in Figure 6a, there was almost no change in the bare chip capacitance, which proved the stability of our fabricated capacitor. In addition, with an increase in temperature, the capacitor-based biosensor injected with a similar concentration of glucose sample shows a decreasing capacitance, such a phenomenon is mainly due to the higher temperature that gives more energy to the glucose molecules, leading to more active molecule movements, and thus a higher viscosity within a fixed volume of glucose sample. Therefore, a lower dielectric constant can be achieved and, finally, results in a decrease in capacitance as long as the temperature is increasing. Furthermore, the slope value (|tan θ|) and maximum variation (ΔC) of the capacitance fitting curve at different glucose concentrations are calculated, as shown in Figure 6b. The |tan θ| and ΔC are both decreasing when the glucose concentration is increasing, while the temperature is also increasing. It can be concluded that a higher concentration of the glucose sample suffers less from temperature influence as comparing with a lower glucose concentration. Such a phenomenon is mainly due to the fact that the higher concentration glucose sample possesses more glucose molecules within a fixed sample volume, and therefore the glucose molecules have limited space to be active, resulting in a relatively lower viscous effect and a smaller variation in |tan θ| and ΔC. Therefore, consistent with our original design intention, it is necessary to design a temperature sensor before measuring capacitance to result in more accurate measurements of glucose concentration.

**Figure 7:**
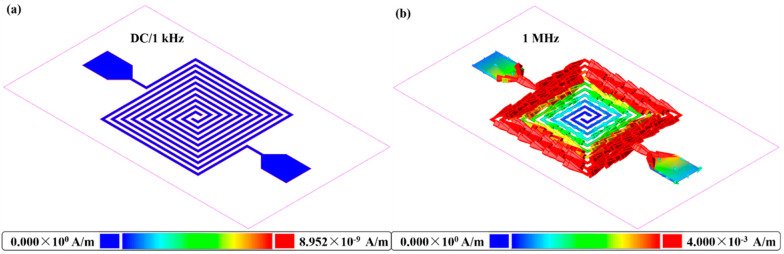
Current density diagram of the capacitor-based biosensor: (**a**) At DC and 1 kHz signal frequency; (**b**) at 1 MHz signal frequency.

Figure 7 shows the current density of the proposed capacitor-based biosensor. As shown in Figure 7a, a dark blue color can be observed for the proposed biosensor at both DC and 1 kHz, even though the maximum current density at this scale is only 8.952 × 10^−9^ A/m, indicating that a few of the input signals couple between the conductor gaps. However, the simulation results of the current density for the proposed biosensor at 1 MHz shows a dark red color with a maximum value of 4.000 × 10^−3^ A/m at the scale bar, as shown in Figure 7b, indicating that the input signal not only concentrates on the conductor itself, but also has strong coupling between the conductor gaps. Under such a condition, the tested glucose solution that flows in the conductor gap could be in full contact with the electric field at stable ambient conditions in the microfluidic cavity, providing stable mathematical statistics of R^2^, RSD, and LOD. Moreover, signal with higher frequency possesses smaller coverage area as compared with lower frequencies such as DC and 1 kHz, thus, smaller coverage area benefits from fewer ambient noise interferences, which also verifies the excellent properties of R^2^, RSD, and LOD of a 1 MHz biosensor. Among these three frequencies, the measurement results of 1 MHz show relatively excellent linearity, sensitivity, LOD, and RSD. Therefore, we can consider this frequency in practical glucose biosensor applications. A performance comparison between this study and previously reported studies is summarized in Table 2 in terms of sensor structure, sensitivity (in molar values), detection range (in molar values), detection limit (in molar values), temperature calibration, response time, and quantitative measurement. It can be inferred that the proposed work is excellent, especially for the functions of temperature calibration, quick response time, and quantitative measurement. Although the microwave biosensor mentioned in [36] also achieved the abovementioned functions, it suffered from relatively low sensitivity and higher sample consumption. Moreover, the proposed biosensor in this study possessed a wide detection range which covered glucose measurements in the application of diabetes testing, as well as applications in some fruits and sweet foods. In regard to the detection limit, a moderate performance was obtained with a value of 0.1636 mmol/L at 1 MHz; such a value was not excellent enough as compared with electrochemical, SERS, optoelectronic, and LFA biosensors. Furthermore, since the proposed biosensor was in different sensitivity units as comparing with the reported electrochemical, SERS, optoelectronic, and LFA biosensors, proper comparisons could not be made. Few of the reported studies focused on a capacitor-based biosensor; among the studies, humidity sensors were quite popular. However, this study focused on a biosensor test based on the structure of the capacitor, providing a new method for glucose testing.

## 6. Conclusions

Our study investigated a resistor-based temperature sensor and capacitor-based biosensor combining with a PDMS microfluidic channel, functioning for real-time, quantitative, and temperature-calibrated glucose detection. The resistor and capacitor were designed with symmetrical meandering and intertwined structure, respectively, so that compact chip size of integrated sensors could be achieved. Moreover, we managed to apply a PDMS microfluidic channel with fixed shape, fixed volume, and fixed test position, and integrated it with the metal structure of the glass substrate. The results from this study suggest that the resistor-based and capacitor-based biosensor responds rapidly and linearly to a range of temperatures (25–100 °C) and glucose solution concentrations (25–1000 mg/dL) with good sensitivity, excellent stability, and low cost. Owing to the outstanding performance of our developed temperature-calibrated glucose biosensor, a new method is proposed for clinical detection of diabetes in early stage, which is of great medical significance for early detection of diabetes.

## Figures and Tables

**Table 1:** Performances of the different measured capacitance for the bare chip and under DI water for the PDMS cavity-based DC/1 kHz/1 MHz capacitor-based biosensor.

^1^ S.F	Item	Capacitance (pF/nF)					RSD (%)
		1st Test	2nd Test	3rd Test	^2^ Mean	Mean ± RSD (^3^ *C*_av_)	
DC	Bare Chip	28.023 nf	28.99 nf	28.877 nf	28.630 nf	28.63 nf ± 1.847	1.847
	DI water	938.945 nf	957.672 nf	980.598 nf	959.072 nf	959.072 nf ± 2.175	2.175
1 kHz	Bare Chip	3.224 pf	3.262 pf	3.244 pf	3.243 pf	3.243 pf ± 0.586	0.586
	DI water	64.375 nf	65.174 nf	66.37 nf	65.306 nf	65.306 nf ± 1.537	1.537
1 MHz	Bare Chip	2.424 pf	2.393 pf	2.405 pf	2.407 pf	2.407 pf ± 0.649	0.649
	DI water	23.483 pf	23.543 pf	23.528 pf	23.518 pf	23.518 pf ± 0.133	0.133

^1^ S.F, signal frequency; ^2^ mean = average of the three experiments; ^3^ *C*_av_, final average capacitance.

**Table 2:** Performance comparison with other previously reported methods.

Refs.	Sensor Structure	Sensitivity		Detection Range	Detection Limit	Temperature Calibration	Real Time	Quantitative Test
[37]	Electrochemical biosensor	65.6 μA/mmol·L^−1^·cm^2^		0.0003–2.1 mmol/L	0.3 μmol/L	No	No	No
[38]	Electrochemical biosensor	1.41 μA/mmol·L^−1^		0.1–25 mmol/L	25 μmol/L	No	No	Yes
[39]	Electrochemical based nonenzymatic biosensor	1467.32 μA/mmol·L^−1^·cm^2^		0.005–5.89 mmol/L	0.012 μmol/L	No	No	No
[36]	Microwave biosensor	0.00144 dB/mmol·L^−1^		0–22.22 mmol/L	-	Yes	Yes	Yes 3.9 μL
[40]	SERS biosensor	2350 a.u./mol·L^−1^		0–1 mol/L	0.01 mol/L	No	No	No
[41]	Optoelectronic biosensor	13.6 uA mM^−1^ cm^−2^		0–11 mmol/L	0.015 mmol/L	No	No	No
[42]	LFA biosensor	-		0−5.56 mmol/L	0.0128 mmol/L	No	No	No
This work	Capacitor-based biosensor	DC	2.574 nF/mmol·L^−1^	1.39–55.56 mmol/L	3.735 mmol/L	Yes	Yes	Yes 1.806 μL
		1kHz	0.864 nF/mmol·L^−1^		0.9847 mmol/L			
		1MHz	0.198 pF/mmol·L^−1^		0.1636 mmol/L			

## Data Availability

Not applicable.

## Supplementary Materials

The following are available online at https://www.mdpi.com/article/10.3390/bios11120484/s1, Video S1: Measurement Step: LCR Meter + Proposed Biosensor + Syringe + Tube + Connection Line + Beaker.

## References

[1] Li H.Y., Lin H.Y., Lv W.X., Gai P.P., Li F. (2020). Equipment-free and visual detection of multiple biomarkers via an aggregation induced emission luminogen-based paper biosensor. Biosens. Bioelectron..

[2] Bai Y., Xu T., Zhang X. (2020). Graphene-Based Biosensors for Detection of Biomarkers. Micromachines.

[3] Gou Y., Liu J., Sun C., Wang P., You Z., Ren D. (2021). Inertial-Assisted Immunomagnetic Bioplatform towards Efficient Enrichment of Circulating Tumor Cells. Biosensors.

[4] Kim J., Campbell A.S., Wang J. (2018). Wearable non-invasive epidermal glucose sensors: A review. Talanta.

[5] Zatterale F., Longo M., Naderi J., Raciti G.A., Desiderio A., Miele C., Beguinot F. (2020). Chronic Adipose Tissue Inflammation Linking Obesity to Insulin Resistance and Type 2 Diabetes. Front. Physiol..

[6] Katakami N. (2018). Mechanism of Development of Atherosclerosis and Cardiovascular Disease in Diabetes Mellitus. J. Atheroscler. Thromb..

[7] Khan N.I., Maddaus A.G., Song E. (2018). A Low-Cost Inkjet-Printed Aptamer-Based Electrochemical Biosensor for the Selective Detection of Lysozyme. Biosensors.

[8] Govindasamy M., Wang S.F., Subramanian B., Ramalingam R.J., Al-Lohedan H., Sathiyan A. (2019). A novel electrochemical sensor for determination of DNA damage biomarker (8-hydroxy-2′-deoxyguanosine) in urine using sonochemically derived graphene oxide sheets covered zinc oxide flower modified electrode. Ultrason. Sonochem..

[9] Wang Y.H., Wang F.T., Han Z.W., Huang K.J., Wang X.M., Liu Z.H., Wang S.Y., Lu Y.F. (2020). Construction of sandwiched self-powered biosensor based on smart nanostructure and capacitor: Toward multiple signal amplification for thrombin detection. Sens. Actuators B Chem..

[10] Meng W., Wen Y., Dai L., He Z., Wang L. (2018). A novel electrochemical sensor for glucose detection based on Ag@ZIF-67 nanocomposite. Sens. Actuators B Chem..

[11] Manavalan S., Ganesamurthi J., Chen S.M., Veerakumar P., Murugan K. (2020). A robust Mn@FeNi-S/graphene oxide nanocomposite as a high-efficiency catalyst for the non-enzymatic electrochemical detection of hydrogen peroxide. Nanoscale.

[12] Jahn I.J., Grjasnow A., John H., Weber K., Popp J., Hauswald W. (2021). Noise Sources and Requirements for Confocal Raman Spectrometers in Biosensor Applications. Sensors.

[13] Tao W., Song Y., Singhal N., McGoverin C., Vanholsbeeck F., Swift S. (2021). A novel optical biosensor for in situ and small-scale monitoring of bacterial transport in saturated columns. J. Environ. Manag..

[14] Williams R.M., Lee C., Heller D.A. (2018). A Fluorescent Carbon Nanotube Sensor Detects the Metastatic Prostate Cancer Biomarker uPA. ACS Sens..

[15] Severi C., Melnychuk N., Klymchenko A.S. (2020). Smartphone-assisted detection of nucleic acids by light-harvesting FRET-based nanoprobe. Biosens. Bioelectron..

[16] Selvarajan R.S., Rahim R.A., Majlis B.Y., Gopinath S.C.B., Hamzah A.A. (2020). Ultrasensitive and Highly Selective Graphene-Based Field-Effect Transistor Biosensor for Anti-Diuretic Hormone Detection. Sensors.

[17] Tsang D.K.H., Lieberthal T.J., Watts C., Dunlop I.E., Ramadan S., Hernandez A.E.d.R., Klein N. (2019). Chemically Functionalised Graphene FET Biosensor for the Label-free Sensing of Exosomes. Sci. Rep..

[18] Mei J., Li Y.-T., Zhang H., Xiao M.-M., Ning Y., Zhang Z.-Y., Zhang G.-J. (2018). Molybdenum disulfide field-effect transistor biosensor for ultrasensitive detection of DNA by employing morpholino as probe. Biosens. Bioelectron..

[19] Baldacchini C., Montanarella A.F., Francioso L., Signore M.A., Cannistraro S., Bizzarri A.R. (2020). A Reliable BioFET Immunosensor for Detection of p53 Tumour Suppressor in Physiological-Like Environment. Sensors.

[20] Pohanka M. (2018). Piezoelectric biosensor for the determination of Tumor Necrosis Factor Alpha. Talanta.

[21] Zhang W.L.H., Zhang L.L., Gao H.L., Yang W.Y., Wang S., Xing L.L., Xue X.Y. (2018). Self-Powered Implantable Skin-Like Glucometer for Real-Time Detection of Blood Glucose Level In Vivo. Nano-Micro Lett..

[22] Ebrahimi A., Scott J., Ghorbani K. (2020). Microwave reflective biosensor for glucose level detection in aqueous solutions. Sens. Actuators A-Phys..

[23] Bahar A.A.M., Zakaria Z., Arshad M.K.M., Isa A.A.M., Dasril Y., Alahnomi R.A. (2019). Real Time Microwave Biochemical Sensor Based on Circular SIW Approach for Aqueous Dielectric Detection. Sci. Rep..

[24] Alibakhshikenari M., Virdee B.S., Shukla P., Parchin N.O., Azpilicueta L., See C.H., Abd-Alhameed R.A., Falcone F., Huynen I., Denidni T.A. (2020). Metamaterial-Inspired Antenna Array for Application in Microwave Breast Imaging Systems for Tumor Detection. IEEE Access.

[25] Yu X., Chen X.D., Ding X., Zhao X. (2018). A High-Stability Quartz Crystal Resonator Humidity Sensor Based on Tuning Capacitor. IEEE Trans. Instrum. Meas..

[26] Liang J.G., Wang C., Yao Z., Liu M.Q., Kim H.K., Oh J.M., Kim N.Y. (2018). Preparation of Ultrasensitive Humidity-Sensing Films by Aerosol Deposition. ACS Appl. Mater. Interfaces.

[27] Chappanda K.N., Chaix A., Surya S.G., Moosa B.A., Khashab N.M., Salama K.N. (2019). Trianglamine hydrochloride crystals for a highly sensitive and selective humidity sensor. Sens. Actuators B Chem..

[28] Lee H.-J., Yook J.-G. (2014). Recent research trends of radio-frequency biosensors for biomolecular detection. Biosens. Bioelectron..

[29] Bababjanyan A., Melikyan H., Kim S., Kim J., Lee K., Friedman B. (2010). Real-Time Noninvasive Measurement of Glucose Concentration Using a Microwave Biosensor. J. Sens..

[30] Kumar A., Wang C., Meng F.Y., Zhou Z.L., Zhao M., Yan G.F., Kim E.S., Kim N.Y. (2020). High-Sensitivity, Quantified, Linear and Mediator-Free Resonator-Based Microwave Biosensor for Glucose Detection. Sensors.

[31] Murji R., Deen M.J. (2002). A scalable meander-line resistor model for silicon RFICs. IEEE Trans. Electron Devices.

[32] Lall M.P.P., Dorf R.C. (1997). Resistors. Electrical Engineering Handbook.

[33] Endres H.E., Drost S. (1991). Optimization of the Geometry of Gas-Sensitive Interdigital Capacitors. Sens. Actuators B Chem..

[34] Yoon G. (2011). Dielectric properties of glucose in bulk aqueous solutions: Influence of electrode polarization and modeling. Biosens. Bioelectron..

[35] Topsakal E., Karacolak T., Moreland E.C. Glucose-dependent dielectric properties of blood plasma. Proceedings of the 2011 3th URSI General Assembly and Scientific Symposium.

[36] Jang C., Park J.K., Lee H.J., Yun G.H., Yook J.G. (2020). Non-Invasive Fluidic Glucose Detection Based on Dual Microwave Complementary Split Ring Resonators with a Switching Circuit for Environmental Effect Elimination. IEEE Sens. J..

[37] Yoon H., Nah J., Kim H., Ko S., Sharifuzzaman M., Barman S.C., Xuan X., Kim J., Park J.Y. (2020). A chemically modified laser-induced porous graphene based flexible and ultrasensitive electrochemical biosensor for sweat glucose detection. Sens. Actuators B Chem..

[38] Cao L., Han G.-C., Xiao H., Chen Z., Fang C. (2020). A novel 3D paper-based microfluidic electrochemical glucose biosensor based on rGO-TEPA/PB sensitive film. Anal. Chim. Acta.

[39] Ahmad R., Khan M., Mishra P., Jahan N., Ahsan M.A., Ahmad I., Khan M.R., Watanabe Y., Syed M.A., Furukawa H. (2021). Engineered Hierarchical CuO Nanoleaves Based Electrochemical Nonenzymatic Biosensor for Glucose Detection. J. Electrochem. Soc..

[40] Sung C.-J., Chao S.-H., Hsu S.-C. (2021). Rapid Detection of Glucose on Nanostructured Gold Film Biosensor by Surface-Enhanced Raman Spectroscopy. Biosensors.

[41] Yang W., Xu W., Zhang N., Lai X., Peng J., Cao Y., Tu J. (2020). TiO_2_ nanotubes modified with polydopamine and graphene quantum dots as a photochemical biosensor for the ultrasensitive detection of glucose. J. Mater. Sci..

[42] Ki H., Jang H., Oh J., Han G.-R., Lee H., Kim S., Kim M.-G. (2020). Simultaneous Detection of Serum Glucose and Glycated Albumin on a Paper-Based Sensor for Acute Hyperglycemia and Diabetes Mellitus. Anal. Chem..

